# Zymotic Disease in London

**Published:** 1902-01-18

**Authors:** 


### Zymotic Disease in London.

At a time when attention is being largely concentrated upon a single one of the group of zymotic diseases, namely, small-pox—the most loathsome, perhaps, but by no means the one which, taking any series of years, makes the largest impression on the death-rate—it may be well to draw attention to the proportional importance of the several members of this group. In the last of the admirable annual reports issued by Mr. Shirley Murphy, the medical officer of health of the county of London, we find that during the year 1900 no less than 10,046 persons died in London from the eight "principal zymotic diseases," small-pox, measles, scarlet fever, diphtheria, typhus, enteric, and ill-defined fevers, and diarrhœa, being a rate equal to 2·25 per 1,000 living, which, however, is a little less than the average for some years past.

If, however, to avoid accidental error due to local and temporary outbreaks of disease, we take the average for the 10 years 1891 to 1900, and if we sort out these diseases into two groups, one containing all those for which some degree of hospital accommodation is provided, and the other those which have to be dealt with at home, wherever they occur, we see what a small fringe of the whole problem presented by zymotic disease in London is dealt with by our public hospitals. Small-pox, scarlet fever, diphtheria, typhus, enteric, and simple continued fever caused altogether a mortality of 0·81 per 1,000 living, while the following three diseases, measles, whooping cough, and diarrhœa, together caused a mortality of 1·86 per 1,000 living, or considerably more than twice the death-rate resulting from all the rest put together. Notwithstanding also that, compared with the previous decennium, measles has diminished while diphtheria has considerably increased, measles yet shows the higher death-rate; while, comparing it with scarlet fever, measles kills on the average four times as many people as die from this much-dreaded disease.

Whooping cough, again, is very nearly as fatal a malady as measles; it stands level with diphtheria, notwithstanding that the latter has increased nearly 100 per cent. as compared with the previous decennium, and is far far ahead of the scarlet and enteric fevers about which we hear so much. Diarrhœa is the most fatal of all the principal zymotic diseases, carrying off half as many again as are killed by diphtheria and more than twice as many as all the scarlet fevers and enteric fevers put together. With these facts before us—the steady growth of the great hospitals for the treatment of infectious diseases; the fact that notwithstanding the immense expense which their maintenance runs to, these hospitals do but receive at the outside about 70 per cent. of the total number of the class of cases for which they are provided; and, lastly, the fact that the zymotic diseases for which no such provision is made, cause a good deal more than twice as high a death-rate as arises from the whole of those notifiable diseases for which hospitals are provided put together; we come to the very serious question, How is this vast and unmanageable mass of zymotic disease to be controlled? We will not pretend to lay down laws, or to speak positively on the subject, but we will say at once that a careful study of Mr. Shirley Murphy's report is sufficient to show that whatever may be done in the direction of hospital treatment—and there are many of these poor children who are eminently fitted for hospital treatment—a great deal else will have to be effected, especially in the direction of altering the home conditions which lead to the prevalence and fatality of these diseases if any serious impress is to be made upon them.

There are certain diseases which may attack anyone, and which, when the prevailing type is severe, may put anyone's life in peril, even amid good surroundings. But such diseases as measles and whooping cough are not of this class, neither of them being very serious matters when they occur amid good surroundings, and are treated with reasonable care. Nothing is more striking than to note how greatly the death-rate from these maladies varies in the different districts of the metropolis; in other words how markedly unhealthy surroundings tell in determining their fatality.

Taking the average of ten years, measles caused a death-rate in Holborn, Clerkenwell, St. Luke, Shoreditch, Bethnal Green, St. George-in-the-East, Limehouse, St. Saviour Southwark, St. George Southwark, and in Bermondsey, which was in all cases more than three times as great as in the more favoured district of Hampstead.

Turning, then, to the next disease on our list, we find that in every one of these same parishes, as well as in some others, the mortality from whooping cough was more than twice as great as in more healthy areas, and finally we find diarrhœa also selecting many of these unfortunate parishes as their special homes.

Much is being done in the way of isolation, but if any definite grip is to be gained upon the whole mass of zymotic disease as it prevails in London, it must be by modifying the conditions of life in those districts where these diseases most prevail.

